# Organohalogenated Substances and Polycyclic Aromatic Hydrocarbons in Fish from Mediterranean Sea and North Italian Lakes: Related Risk for the Italian Consumers

**DOI:** 10.3390/foods11152241

**Published:** 2022-07-27

**Authors:** Giacomo Mosconi, Federica Di Cesare, Francesco Arioli, Maria Nobile, Doriana E. A. Tedesco, Luca M. Chiesa, Sara Panseri

**Affiliations:** 1 Department of Veterinary Medicine and Animal Science, University of Milan, Via dell’Università 6, 26900 Lodi, Italy; giacomo.mosconi@unimi.it (G.M.); federica.dicesare@unimi.it (F.D.C.); luca.chiesa@unimi.it (L.M.C.); sara.panseri@unimi.it (S.P.); 2 Department of Environmental Science and Policy, University of Milan, Via G.Celoria 2, 20133 Milan, Italy; doriana.tedesco@unimi.it

**Keywords:** persistent organic pollutants, gilthead seabream, European seabass, agone, GC-MS/MS, food safety, intake evaluation

## Abstract

The primary source of persistent organic pollutant (POP) exposure is food, especially fish. European seabass (*Dicentrarchus labrax*) and gilthead seabream (*Sparus aurata*) are among the most eaten sea fish in Italy. Fish from lakes in Northern Italy, such as agone (Alosa agone), represent niche consumption for most people, but possibly constitute a much larger percentage of overall consumption volume for local residents. This study dealt with the presence of POPs in the above-mentioned fish species via GC-MS/MS analysis. None of the analytes for which maximum limits are in place showed concentrations above those limits. Moreover, none of the substances without maximum limits exceeded the provisional tolerable daily intake (PTDI) when given, nor did they exceed the more general values considered safe, even for 99th percentile consumers.

## 1. Introduction

The presence and distribution of persistent organic pollutants (POPs), such as polychlorinated biphenyls (PCBs), organochlorine pesticides (OCPs), polybrominated diphenyl ethers (PBDEs), and polycyclic aromatic hydrocarbons (PAHs), is considered a worrisome environmental issue due to their bioaccumulation, persistence, and toxicity [1]. Consequently, there is a crucial need to understand the levels and trends, and their impacts on marine and lake fish, to evaluate the potential threat to consumer health. Fish represent an essential ecological component of aquatic ecosystems and can accumulate to many different lipophilic contaminants [2,3,4]. Although several monitoring studies have shown that halogenated compounds, such as PCBs and PBDEs, have been decreasing in marine ecosystems over the past 10 years [5], these contaminants are still detected in fish from various FAO areas [6,7]. Exposure to these pollutants can occur through the skin and through respiration, but mainly, about 90% occurs through the diet, in which food of animal origin, particularly fish, plays a major role [8]. OCPs are commonly categorized as endocrine-disrupting chemicals (EDCs) and are known to increase hormone-related cancer risk [9]. Additionally, OPCs exhibit many toxic effects on development, immunological function, and animal reproduction [10]. Moreover, epidemiological works confirmed a positive correlation between high concentrations of OCPs and the incidence of cardiovascular diseases, hypertension, and other diseases in humans [11]. Aldrin, endrin, heptachlor, dichlorodiphenyltrichloroethane (DDT), and other OCPs were listed in the initial “dirty dozen” of POPs after the Stockholm Convention in 2001. In 2009, hexachlorocyclohexane (HCH) isomers such as α-HCH, β-HCH, and γ-HCH were added to the list [12]. PBDEs, well known as flame-retardants, are used in a number of different products, including paints, textiles, furniture, electronic circuit boards, and plastics. Toxicological studies have proven a positive correlation between high levels of PBDE exposure with the onset of thyroid homeostasis disruption, reproductive disorders, neurotoxic effects, and cancer [13].

PCBs include 209 congeners, industrially produced as technical mixtures for dielectric fluids, organic diluents, and flame retardants. Although their production and use were prohibited in the 1977s in the USA and in the 1980s in most European Countries, they are still present in the environment, due to their long persistence and bioaccumulation properties through different food chains. These compounds can exhibit toxic effects on the nervous, immune, endocrine, and reproductive systems [14].

PAHs are derived from both anthropogenic activities (incinerators, industrial processes, motor vehicles, combustion of wood and fossil fuels, oil spills, etc.) and natural sources (incomplete combustion of organic matter and pyrolysis) [15]. Many studies have reported the mutagenic and carcinogenic effects of PAHs and chronic metabolite exposure as described by the European Food Safety Authority [16]. Indeed, the International Agency for Research on Cancer has classified several high molecular weight PAHs as recognized (class 1), probable (Class 2A), or possible (class 2B) human carcinogens [17].

Fish are fundamental to the human diet for their nutritional properties. Several studies have shown a relationship between fish consumption and reduced risk of heart disease, cardiac arrhythmias, and diabetes due to the high percentage of n3PUFA they contain [18]. Among sea fish, gilthead seabream (*Sparus aurata*) and European seabass (*Dicentrarchus labrax*) are two of the most consumed in Italy (14.8% of the total consumption volume) by everyone from young children to the elderly [19]. More than 20% of total EU aquaculture production is represented by these two species [20]. Additionally of note, lakes are important for POP bioaccumulation studies, due to their long residence time in closed basins, as well as the increasing anthropological activities of residential populations [21].

In literature, there are few recent studies on the detection of POPs in sea fish or in lake fish (Table 1), and none have dealt with both sea fish and lake fish in order to more comprehensively analyze the risks faced by different consumers, as we had in a recent article dealing with PFASs [22].

Thus, the present study aimed to measure the concentrations of OCPs, PCBs, PAHs, and PBDEs in the fillets of different fish species from both sea and lake to evaluate the possible risks to the Italian consumer.

## 2. Materials and Methods

### 2.1. Chemicals and Reagents

All solvents for pesticide residue analysis (Pestanal) were from Merck (Darmstadt, Germany). The PCB congener mixture (PCB 28, PCB 52, PCB 101, PCB 138, PCB 153, PCB 180) and PCB 209 as an internal standard (IS), as well as the PBDE mixture (PBDE 28, PBDE 33, PBDE 47, PBDE 99, PBDE 100, PBDE 153, PBDE 154) and 3-fluoro-2,2,4,4,6-pentabromodiphenyl ether (FBDE) as IS, were from AccuStandard (New Haven, CN, USA). α-HCH, β-BHC, lindane, heptachlor, heptachlor epoxide, aldrin, endrin, endosulphan I, endosulphan II, endosulphan sulphate, trans chlordane, 4,4′-DDE, 4,4′-DDT, 2,4′-DDT, 4,4′-DDD, Chrysene, Benz(a)anthracene, Benzo(b)fluoranthene, and Benzo(a)pyrene were obtained from Restek (Bellefonte, PA, USA).

### 2.2. Standard Solutions

Working solutions were prepared in hexane from different stock solutions containing a mix of standards and stored at −20 °C.

### 2.3. Sample Collection

Seventy-four sea fish were collected: thirty-five European seabasses (*Dicentrarchus labrax*) and thirty-nine gilthead seabreams (*Sparus aurata*) (weight 450–550 g, age 18–22 months for both sea species) from Italy, Croatia, Greece, Malta, and Turkey. Seventy-nine agones (*Alosa agone*) (weight 150–250 g) were collected from two of the most representative lakes in Northern Italy (Lake Garda and Lake Como).

### 2.4. Extraction and Clean-Up Protocol

The extraction of chlorinated, brominated compounds and PAHs from the fish fillet was carried out according the QuEChERS approach described by Chiesa et al. [34,35]. A 2 g sample of fish muscle was extracted by a QuEChERS Citrate extraction tube. The two different ISs, FBDE and PCB 209, were added at concentrations of 1 ng g^−1^ and 10 ng g^−1^, respectively, followed by the addition of a mixture (4:1 *v*/*v*) of hexane/ethyl acetate (10 mL), vortexed and centrifuged for 10 min at 5000× *g* at 4 °C. Then, the supernatant was purified by a clean-up tube (Z-Sep), and after evaporation of the extract, it was dissolved in 1 mL of hexane.

### 2.5. GC-MS Analysis

The analysis was performed by triple quadrupole mass spectrometry in electronic impact (EI) mode. A GC Trace 1310 chromatograph through a fused-silica capillary column Rxi-XLB (30 m 0.25 mm i.d., 0.25 mm film thickness, Restek, Bellefonte, PA, USA) was coupled to a TSQ8000 triple quadrupole mass detector (Thermo Fisher Scientific, Palo Alto, CA, USA). The oven temperature program and all mass parameters were described by Chiesa et al. [34]. The detector operated in selected reaction monitoring mode (SRM), detecting from two to four transitions per analyte, reported with the collision energies in Table 2. Xcalibur^TM^ and Trace Finder 3.0 (Thermo Fisher Scientific) were the softwares used for instrument control and data analysis.

### 2.6. Validation of the Method

Validation was assessed following the SANTE Guidance 11312/2021 [36].

The absence of interference was verified by the lack of peaks with a signal-to-noise ratio S/N > 3 at the expected retention times of all analytes. The selectivity was evaluated on extracted blank fish samples for the different species. For linearity, 2 g of blank fish were spiked to cover the concentration range from 0.5 to 50 ng g^−1^ (0.5, 1, 5, 10, and 50 ng g^−1^) for all the analytes. The limit of quantification (LOQ) of the methods was the lowest spiked level meeting the requirements of recovery within the range of 70–120% and an RSD ≤ 20%. Recoveries were calculated at LOQ for all compounds. The repeatability (evaluated as the relative standard deviation, RSD) was calculated on 6 replicates at the same fortification level.

### 2.7. Statistical Analysis

Statistical analyses were done by GraphPad InStat (GraphPad Software, Inc version 3.10) software. Non-parametric valuation was conducted, after which, a Kolmogorov-Smirnov Test revealed that the data were not normally distributed. A non-parametric Mann–Whitney Rank Sum Test was used to compare the median values of two datasets, while Kruskal–Wallis One Way analysis was used to compare the medians of more than two datasets (*p* was set at 0.05).

## 3. Results and Discussions

### 3.1. Validation

Limits of Quantification (LOQs) were 0.50 ng g^−1^ for PCBs, PBDEs, and OCPs, and 1.0 ng g^−1^ for PAHs. All the validation parameters reported in Table 3 satisfied the SANTE 11312/2021 Guidance [36].

### 3.2. Occurrence of NDL-PCBs, OCPs, PBDEs, and PAHs in Sea and Lake Fish

All the results are summarized in Table 4.

The results concerning PCBs are expressed in ng g^−1^ wet weight, as their maximum limits (MLs) are expressed in the muscle of fish and fishery products and the muscle of wild-caught freshwater fish by Commission Regulation (EU) No 1259/2011 [37]. All samples showed the presence of PCBs below the quantification limit for at least one congener, but one sample from the sea and four samples from the lake did not show traces of PCB 28, and only one sample from Lake Como did not show traces for PCB 52. Quantifiable concentrations for the sum of the six non-dioxin-like PCBs (NDL-PCBs), both from marine and lake environments, were found in all the samples, with the highest prevalence for PCB 138 quantified in 87.5% of the samples from Lake Como. Statistically significant differences were found between European seabass and gilthead seabream for PCB 153 (*p* < 0.05) and the sum of the six indicators (*p* < 0.05), with the highest concentrations in the gilthead seabream samples. As regards agone, Lake Como showed higher concentrations than Lake Garda (*p* < 0.0001).

Among PBDEs, the congener BDE-47 was always found in the freshwater samples. Each freshwater sample showed at least traces of this compound, and the highest concentration of any sample was from freshwater (5.72 ng g^−1^ w.w.). BDE-99, which in the EFSA report [38] shows a potential health concern with respect to current dietary exposure in the EU, was found in traces in 86.2% and in a quantifiable amount in 19.0% of freshwater samples, with the highest concentration in a sample from Lake Como (1.34 ng g^−1^ w.w.). For this class of compounds, there were no statistically significant differences between the different FAO zones and species for the marine samples; in the lake environment the concentrations of three different compounds, namely BDE-47, BDE-99, and BDE-100, were found to be higher in Lake Como than in Lake Garda, with a statistically significant difference (*p* < 0.0001, *p* < 0.05 and *p* < 0.0001 respectively).

Among compounds of the OCP class, the one most found in a quantifiable amount was the sum of DDT and metabolites, with 100% of the samples from the lake environment above LOQ, while for the marine species, there was a quantification rate of 94.3% for sea bass and 84.6% for sea bream. The highest quantified value was found in a sample from Lake Como, with a concentration of 32.73 ng g^−1^ w.w. The sum of the three HCH isoforms was quantified in 25.7% of the sea bass and 23.1% of the sea bream; in contrast, quantifiable concentrations were found in only 12.5% of the samples from Lake Como. The highest value of 1.66 ng g^−1^ w.w. was found in a sample of sea bass. The concentration for the sum of DDT and its metabolites was found higher in the sea bass in contrast with sea bream (*p* < 0.02) and in the samples from Lake Como (*p* < 0.0005). Moreover, in lake environments, significant differences were found in the concentrations of Trans Chlordane and Endosulfan I (*p* < 0.002, *p* < 0.005 respectively), with the higher values found in the samples from Lake Como.

Regarding PAHs, only 5.71% of the sea bream exceeded the LOQ for Benz(a)anthracene while Benzo(b)fluoranthene showed values higher than the LOQ in 2.86%, 7.69%, 8.51%, and 15.63% of the sea bass, sea bream, Lake Garda’s and Lake Como’s samples, respectively.

### 3.3. Risk Characterization

#### 3.3.1. Substances with Maximum Limits: NDL-PCBs

Among the 209 possible PCB congeners, only 12 compounds of dioxin-like PCBs have a mechanism of toxicity comparable to that of dioxins, due to their ability to bind the aryl hydrocarbon receptor [39]. The monitoring of PCB contamination in food, moreover, is also based on the determination of six congeners targeted: namely, the NDL-PCBs congeners n. 28, 52, 101, 138, 153, and 180, which represent 50% of all NDL-PCBs in food [40]. The highest concentration of the six NDL-PCBs, related to both sea and lake fish, was 36 ng g^−1^ w.w.; therefore, no sample exceeded the MLs for this class of compounds (75 ng g^−1^ for muscle meat of fish and 125 ng g^−1^ for muscle meat of wild-caught freshwater, respectively) [37].

#### 3.3.2. Substances without Maximum Limits

As regards fish consumption in Italy, in a recent work [22], we faced a substantial lack of data about the consumption of niche lake food such as agone, a very typical fish found in north Italian lakes. Briefly, we started from a EUMOFA ‘Case study about the price structure in the supply chain for fresh gilthead seabream in Italy’ [19] to calculate the daily consumption of the lake fish as well as for sea fish. The amount of daily fish consumption was found to be 1.36 g of gilthead seabream, 0.93 g of European seabass, and 0.25 g of agone, per capita. Successively, the Estimated Daily Intake (EDI) of the different analysed substances can be calculated as follows:

EDI = C × DC/BW,(1)

where C is the median concentration of the analyte (or the mean value if higher than the median), DC is the Italian per capita daily fish consumption, and BW is the consumer bodyweight, considered equal to 70 kg. We considered also the 99th percentile estimated seafood consumption, which was calculated as 3.9 times that of the 50th percentile of consumers [41,42]. As a precaution, we used the 99th percentile instead of the 95th, to account for the possible dietary habits of lakeshore inhabitants. Finally, with regard to the concentrations in the lake fish samples, we considered the highest values, i.e., those from Lake Como, with the exception of endosulfan sulphate, lindane, and the sum of PAHs for the risk characterization.

- Polybrominated diphenyl ethers

A report by the European Food Safety Agency [38] indicated ‘Fish and other seafood’ as the ‘dominant food category’ for risk related to PBDEs oral intake. Namely, PBDE-28, 47, 99, 100, 153, 154, 183, and 209 were found relevant for dietary PBDE exposure, and the targets are liver, thyroid hormone homeostasis, and the reproductive and nervous systems. EFSA identified effects on neurodevelopment, which affect behavior, in mice as the critical endpoint, derived the benchmark dose lower 95% confidence limit for a benchmark response of 10% (BMDL10) values, and indicated a margin of exposure higher than 2.5 of no health concern [38]. Of the above-mentioned PBDEs, only the congeners 47, 99, and 100 had a median value higher than the limit of quantification, indicating a possible health concern due to chronic exposure. However, the Contam Panel of EFSA highlighted that appropriate toxicity data were only available for BDE-47, 99, 153, and 209; therefore, a risk characterization is now carried out for congeners 47 and 99, as BDE-100 had not been considered in the report. The PBDE 47 average intakes were 0.29, 0.29, 0.57, and 1.49 ng g^−1^, for gilthead seabream, European seabass, Lake Garda and Lake Como agone, respectively. The intakes for PBDE 99 were 0.25, 0.25, 0.29, and 0.42 ng g^−1^, respectively. As Lake Como PBDEs average concentrations were higher than those of Lake Garda, we considered them for the risk characterization.

If the three fish species are considered as a whole, i.e., if the intakes are added, the average consumer’s EDI for PBDE 47 is 0.015 ng kg^−1^ per day, far lower than the chronic exposure of 1.91 ng kg^−1^ b.w. per day in European countries, considered of no concern by EFSA [38]. With similar reasoning, the average EDI of PBDE 99 is 0.0097 ng kg^−1^ per day lower than the PBDE 99 chronic exposure value of 0.65 ng kg^−1^ per day, which can be considered safe. Even considering the 99th percentile consumers, the EDIs would be 0.058 ng kg^−1^ per day for PBDE 47 and 0.038 for PBDE 99.

- Cyclodienes

The provisional tolerable daily intake (PTDI) of aldrin, expressed as a sum of dieldrin and aldrin, is 100 ng kg^−1^ b.w. per day, based on hepatic tumours in mice [43]. The calculated EDI is 0.0091 ng kg^−1^ b.w. per day, much less than PTDI, also for the 99th percentile (0.035 ng kg^−1^ b.w. per day).

Trans chlordane is, with the Cis- form, one of the two isomers constituting Chlordane, classified by IARC [44] as possibly carcinogenic to humans (Group 2B). Its PTDI is 500 ng kg^−1^ b.w. per day, based on liver toxicity in rats [45]. The calculated EDI of chlordane is 0.012 ng kg^−1^ b.w. per day, which is much less than PTDI, even if the presence of an equal amount of cis-chlordane is hypothesized in fish and the high consumers are considered (0.024 and 0.095 ng kg^−1^ b.w. per day).

Value of Endrin PTDI is 200 ng kg^−1^ [46,47]. The calculated EDIs are 0.033 and 0.13 ng kg^−1^ b.w. per day for average and 99th percentile consumers, respectively.

The safety of endosulfan based on reduced body weights and pathological findings in rats and mice was stated by The Joint FAO/WHO Meeting on Pesticide Residues (JMPR) [48], with an admissible daily intake (ADI) value of 6 × 103 ng kg^−1^ b.w. per day. The EDI values are 0.053 ng kg^−1^ b.w. per day and 0.21 ng kg^−1^ b.w. per day for average and high consumers, respectively.

Since heptachlor epoxide is a major metabolite of heptachlor, these two molecules were considered together. Since average values were always equal the half the LOQ, the sum of the concentrations in the different fish species were all equal to the LOQ. The average EDI was, therefore, 0.019 ng kg^−1^ b.w. per day and 0.074 ng kg^−1^ b.w. per day for 99th percentile consumers. The more recent ADI from JMPR [49] is 100 ng kg^−1^ b.w. per day based on a long-term study on the reproduction of dogs, which is much higher than the calculated EDIs.

- DDT and metabolites

The active principle is commonly p-p’ DDT, but also the metabolites op’ DTT, and the isomers of DDT and DDE are found in the mixture named “DDT complex.” The JMPR [50] reviewed several studies that could also include some metabolites other than pp’ DDT. IARC classifies DDT in Group 2A (probably carcinogenic to humans) based on sufficient evidence in experimental animals, but limited evidence in humans, that DDT exposure can cause non-Hodgkin lymphoma, testicular cancer, and liver cancer [45]. Based on studies of developmental toxicity in rats as the endpoint, the PTDI of DTT complex was estimated as 1 × 104 ng kg^−1^ b.w. per day. The EDI calculation of the DDT complex detected in the fish species considered the median concentration of Lake Como higher than the mean, and the resulting values were 0.18 and 0.69 ng kg^−1^ b.w. per day for mean and high consumers, respectively.

- Halogenated aryl hydrocarbons

HCH exists in isomers α- to ε-. The γ isomer is Lindane, the insecticide belonging to the group of halogenated aryl hydrocarbons. It is carcinogenic to humans (IARC group 1) and is associated with non-Hodgkin lymphoma (IARC group 1) for professional exposures in agriculture [51]. In 2002, the JMPR [52] established an ADI of 500 ng kg^−1^ b.w. per day based on a long-term study on carcinogenicity in rats, in which an increased incidence of periacinar hepatocellular hypertrophy, increased liver and spleen weights, and increased mortality were observed. The EDI of Lindane is 0.012 ng kg^−1^ b.w. per day, which is well below the ADI of 99th percentile consumers (0.047 ng kg^−1^ b.w.). The isomers α-HCH and β-HCH are, however, indicated by the Integrated Risk Information System (IRIS) [53] online database of Environmental Protection Agengy (EPA) as carcinogenetic, with an endpoint constituted by hepatic nodules and hepatocellular carcinomas, and a high slope factor of 6.3 and 1.8 (mg kg^−1^ day^−1^)^−1^, respectively. This leads to a very high Cancer Risk (CR)

CR = SF × LADD,(2)

where LADD is the lifetime average daily dose at the intake of fish previously calculated and SF is the cancer slope factor), whose values are 7.6 × 10^−8^ and 2.1 × 10^−8^ for α-HCH and β-HCH, respectively. As the threshold of concern is one in a million (10^−6^), the calculated values do not indicate a matter of concern after the intake of the fish analysed in this work [54].

- Polycyclic aromatic hydrocarbons

EFSA established a margin of exposure (MOE) approach for the PAH4, i.e., the sum of benzo(a)pyrene, chrysene, benz(a)anthracene, and benzo(b)fluoranthene. The MOE approach was based on the BMDL10 of 3.4 × 10^5^ ng kg^−1^ b.w. per day from studies on carcinogenicity in mice. For PAH4, EFSA stated a MOE equal to or less than 10,000 as the indicator of a risk for consumer health [16]. The EDI for considered fish is 0.027 and 0.10 ng kg^−1^ b.w. per day for mean and high consumers, respectively. The corresponding MOEs are 1.27 × 10^7^ and 3.4 × 10^6^ for average and high consumers, respectively.

## 4. Conclusions

Occurrence of organohalogenated substances and polycyclic aromatic hydrocarbons were studied in both the most consumed sea fish (European seabass and gilthead seabream) and in lake fish from lakes in Northern Italy (agone), which represent niche foods for most, but possibly wide consumption for local residents.

Different classes of environmental contaminants were found in both sea and lake fish, with major contamination mostly for the samples from Lake Como. In particular, the MRLs, where present, have never been exceeded, and for all substances without MRLs, the risk assessment showed no risk for consumers on the basis of estimated daily intake, never exceeding the provisional tolerable daily intake when present or the values considered safe, even for the 99th percentile consumers.

## Figures and Tables

**Table 1:** Recent works present in the literature regarding the detection of POPs in sea or lake fish.

Reference	Analytes	Matrix	Extraction Technique	Instrumental Analysis	Limits of the Method (ng g^−1^)	Application Range Concentration (ng g^−1^)
[23]	15 PCBs, 3 DDTs	Different fishes including gilthead seabream and European seabass	Homogenization, soxhlet extraction with hexane/dichloromethane, clean-up with silica	GC-MS	LOQ = 0.02–0.05 ww *	n.d.–2.86 ww
[24]	7 PCBs, 3 DDTs	Alosa agone	Freeze-drying, soxhlet extraction with n-hexane/acetone, clean-up with Florisil column	GC-ECD	LOQ = 0.1–1 lw **	<1–2944.9 lw
[25]	23 PCBs, 3 DDTs	Alosa agone	Freeze-drying, soxhlet extraction with n-hexane/acetone, clean-up with Florisil column	GC-ECD	LOQ = 0.1–1 lw	10–3500 lw
[26]	9 OCPs, 60-80 PCBs	Different fishes including gilthead sea bream	Freeze-drying, extraction with acetonitrile, Calfo E and Celite 545, clean-up with gel permeation chromaography	GC-MS	LOD = 0.0001–0.0045	n.d.–8.2 ww
[27]	22 OCPs, 6 PCBs, 16 PAHs, 8 PBDEs	European seabass	Homogenization, freeze drying, soxhlet extraction, clean-up with gel permeation chromatography	GC-MS/MS	LOQ = 0.03–20	n.d.–449.6 lw
[28]	6 PCBs, 5 DDTs	Different fishes including agone	Freeze-drying, soxhlet extraction with n-hexane/acetone, clean-up with Florisil column	GC-ECD	LOQ = 0.1–0.5 lw	1.8–650 lw
[29]	4 PAHs	Different matrices including gilthead seabream and European seabass	Homogenization, saponification with ethanolic 2 N potassium hydroxide solution, extraction with n-hexane, SPE clean-up	HPLC-FLD	LOD 0.02–0.08; LOQ 0.06–0.26	<LOQ–0.33
[30]	7 PCBs	Gilthead seabream and European seabass	Freeze-drying, extraction with n-hexane, clean-up wit silica gel and aluminium oxide	GC-MS	LOQ = 0.125	0.11–7.17
[31]	14 PCBs, 6 DDTs	Different fishes including agone	Freeze-drying, soxhlet extraction with n-hexane/acetone, clean-up with Florisil column	GC-ECD	LOD = 1	n.d.–11 lw
[32]	12 PCBs, 7 PAHs, 8 OCPs	Different matrices including seabream	Freeze drying, Quechers extraction with n-hexane/ethyl acetate	GC-MS/MS	LOD 0.02–2.55; LOQ 0.4–17.08	<LOQ–18.76
[33]	6 PCBs, 5 OCPs	European seabass	Freeze-drying, ASE extraction with DCM, clean-up with gel permeation chromatography	GC-HRMS	LOQ = 0.0002–0.00319 ww	0.1–19.9 dw ***

lw *: lipid weight; ww **: wet weight; dw ***: dry weight.

**Table 2:** List of the analysed compounds with their instrument parameters (retention time (RT), polarity, precursor ions, product ions, and collision energies).

Compounds	RT	Polarity	Precursor Ions	Product Ions	Collision Energy
	min		m/z	m/z	V
PCB 28	22.11	Positive	256.0	186.0	20
			257.8	186.1	25
PCB 52	23.56	Positive	291.8	222.0	25
			291.8	257.0	10
PCB 101	28.35	Positive	323.9	254.0	25
			325.8	255.9	25
			327.7	255.9	25
PCB 138	33.27	Positive	359.8	289.9	25
			359.8	324.9	10
PCB 153	34.85	Positive	359.8	289.9	25
			359.8	324.9	10
PCB 180	38.03	Positive	393.8	323.8	25
			393.8	358.9	10
			395.7	323.8	25
PCB 209 (IS)	42.24	Positive	497.6	425.8	26
			497.6	427.7	22
			499.6	427.7	24
PBDE 33	32.03	Positive	246.0	139.0	30
			247.9	139.0	30
			405.8	246.0	10
PBDE 28	32.42	Positive	246.0	139.0	30
			248.0	139.0	30
			407.8	248.0	10
PBDE 47	38.35	Positive	325.8	218.9	24
			327.8	219.0	26
			483.7	325.8	16
			485.7	325.9	14
FBDE (IS)	40.88	Positive	421.8	314.8	30
			423.7	314.9	30
			583.6	423.8	10
PBDE 99	40.94	Positive	403.7	296.8	30
			405.7	296.9	30
			563.6	403.8	20
PBDE 100	41.63	Positive	403.8	296.9	30
			405.8	296.9	30
			563.6	403.8	10
PBDE 153	43.16	Positive	481.7	323.9	30
			483.7	376.8	30
			641.6	481.7	20
PBDE 154	44.24	Positive	483.7	323.8	30
			485.7	325.8	30
			643.6	483.7	20
α-HCH	17.86	Positive	180.9	145.0	10
			180.9	146.0	10
			218.9	183.0	10
β-HCH	19.39	Positive	180.9	145.0	10
			183.0	148.0	10
			218.9	183.0	10
γ-HCH (Lindane)	21.08	Positive	180.9	145.0	10
			183.0	145.0	10
			219.0	183.0	10
Heptachlor	22.30	Positive	271.8	236.9	10
			273.9	236.9	10
			273.9	238.9	10
Aldrin	23.91	Positive	260.9	191.0	30
			262.9	193.0	30
			264.9	192.9	30
Heptachlor Epoxide	26.47	Positive	352.9	262.9	10
			352.9	281.9	10
			354.9	264.9	10
Trans Chlordane	28.36	Positive	372.8	263.9	20
			372.8	265.9	20
			374.8	265.9	20
Endosulfan I	28.60	Positive	372.8	265.9	20
			374.9	266.0	20
			376.8	267.9	20
pp-DDE	30.06	Positive	246.0	176.1	30
			248.0	176.1	30
			317.9	248.0	20
Endrin	31.36	Positive	245.0	173.1	30
			262.9	193.0	30
			280.9	245.0	10
op-DDT	32.25	Positive	235.0	165.1	20
			237.0	165.1	20
Endosulfan II	33.12	Positive	195.0	159.0	10
			240.9	205.9	10
pp-DDD	33.23	Positive	235.0	165.1	20
			237.0	165.1	20
pp-DDT	34.86	Positive	235.0	165.1	20
			237.1	165.1	20
Endosulfan Sulphate	35.57	Positive	271.8	236.9	10
			273.8	236.9	10
			273.8	238.9	10
Chrysene	37.80	Positive	226.1	224.1	30
			228.1	202.2	20
			228.1	226.2	30
Benz(a)anthracene	37.99	Positive	226.1	223.1	30
			226.1	224.1	30
			228.1	202.1	20
			228.1	226.2	30
Benzo(b)fluoranthene	42.05	Positive	250.1	224.1	30
			250.1	248.1	30
			252.1	250.1	30
Benzo(a)pyrene	43.03	Positive	252.1	226.1	30
			252.1	250.2	30
			253.2	227.1	20
			253.2	251.2	30

**Table 3:** LOQs and validation parameters of the analysed compounds.

Compounds	LOQ	RSDr	Recovery
	ng g^−1^	%	%
PCB 28	0.5	11	83
PCB 52	0.5	12	87
PCB 101	0.5	9	88
PCB 138	0.5	12	97
PCB 153	0.5	10	82
PCB 180	0.5	10	88
PCB 209 (IS)	0.5	7	92
PBDE 28	0.5	4	93
PBDE 33	0.5	7	79
PBDE 47	0.5	9	94
PBDE 99	0.5	7	81
PBDE 100	0.5	11	80
PBDE 153	0.5	7	82
PBDE 154	0.5	9	84
FBDE (IS)	0.5	5	90
Aldrin	0.5	12	89
Trans Chlordane	0.5	12	94
Endrin	0.5	10	120
Endosulfan I	0.5	12	95
Endosulfan II	0.5	12	84
Endosulfan Sulphate	0.5	16	75
Heptachlor	0.5	20	120
Heptachlor Epoxide	0.5	14	97
pp-DDE	0.5	14	90
op-DDT	0.5	16	118
pp-DDD	0.5	8	102
pp-DDT	0.5	12	96
α-HCH	0.5	14	119
β-HCH	0.5	12	120
γ-HCH (Lindane)	0.5	14	116
Chrysene	1	3	82
Benz(a)anthracene	1	6	75
Benzo(b)fluoranthene	1	6	78
Benzo(a)pyrene	1	2	75

**Table 4:** Descriptive statistics and the number of not detected, <LOQ, and >LOQ related to the different types of fish. Agone were divided by the lake of origin since there were significant differences between the different lakes. Concentrations are expressed in ng g^−1^.

		European Seabass	Gilthead Seabream	Agone from L. Garda	Agone from L. Como
Compound	N°	35	39	47	32
PCB ICES 6	Average ± SD	3.38 ± 2.63	2.71 ± 3.26	3.56 ± 3.11	10.11 ± 7.94
	n.d.; <LOQ; >LOQ	0; 0; 35	0; 0; 39	0; 0; 47	0; 0; 32
	Median	2.34	1.5	1.78	8.46
	Maximum	12.66	21.08	13.76	36.01
PBDE 28	Average ± SD	0.25 ± 0.00	0.25 ± 0.00	0.25 ± 0.00	0.29 ± 0.10
	n.d.; <LOQ; >LOQ	26; 9; 0	30; 9; 0	16; 31; 0	8; 21; 3
	Median	0	0	0.25	0.25
	Maximum	0.25	0.25	0.25	0.58
PBDE 33	Average ± SD	0.25 ± 0.00	0.25 ± 0.00	0.25 ± 0.00	0.25 ± 0.00
	n.d.; <LOQ; >LOQ	33; 2; 0	33; 6; 0	43; 4; 0	28; 4; 0
	Median	0	0	0	0
	Maximum	0.25	0.25	0.25	0.25
PBDE 47	Average ± SD	0.29 ± 0.10	0.29 ± 0.15	0.57 ± 0.44	1.49 ± 1.08
	n.d.; <LOQ; >LOQ	5; 26; 4	10; 26; 3	0; 25; 22	0; 5; 27
	Median	0.25	0.25	0.25	1.33
	Maximum	0.63	0.88	1.99	5.72
PBDE 99	Average ± SD	0.25 ± 0.00	0.25 ± 0.00	0.29 ± 0.19	0.42 ± 0.28
	n.d.; <LOQ; >LOQ	26; 9; 0	27; 12; 0	8; 37; 2	4; 19; 9
	Median	0	0	0.25	0.25
	Maximum	0.25	0.25	1.34	1.22
PBDE 100	Average ± SD	n.d.	0.25 ± 0.00	0.25 ± 0.00	0.40 ± 0.21
	n.d.; <LOQ; >LOQ	35; 0; 0	37; 2; 0	21; 26; 0	5; 17; 10
	Median	0	0	0.25	0.25
	Maximum	0	0.25	0.25	0.86
PBDE 153	Average ± SD	0.25	0.25 ± 0.00	0.30 ± 0.12	0.40 ± 0.21
	n.d.; <LOQ; >LOQ	34; 1; 0	35; 4; 0	33; 12; 2	21; 7; 4
	Median	0	0	0	0
	Maximum	0.25	0.25	0.63	0.77
PBDE 154	Average ± SD	0.25	n.d.	0.25 ± 0.00	0.25 ± 0.00
	n.d.; <LOQ; >LOQ	34; 1; 0	39; 0; 0	42; 5; 0	28; 4; 0
	Median	0	0	0	0
	Maximum	0.25	0	0.25	0.25
PBDEs Sum	Average ± SD	0.41 ± 0.28	0.43 ± 0.36	1.25 ± 0.68	2.61 ± 1.76
	n.d.; <LOQ; >LOQ	4; 14; 17	5; 18; 16	0; 1; 46	0; 2; 30
	Median	0.25	0.25	1.25	2.33
	Maximum	1.28	1.63	3.59	8.63
Aldrin	Average ± SD	0.25 ± 0.00	0.25 ± 0.00	0.25 ± 0.00	0.25 ± 0.00
	n.d.; <LOQ; >LOQ	32; 3; 0	32; 7; 0	41; 6; 0	29; 3; 0
	Median	0	0	0	0
	Maximum	0.25	0.25	0.25	0.25
Trans Chlordane	Average ± SD	0.40 ± 0.21	0.25 ± 0.00	0.25 ± 0.00	0.29 ± 0.11
	n.d.; <LOQ; >LOQ	28; 4; 3	32; 7; 0	32; 15; 0	11; 18; 3
	Median	0	0	0	0.25
	Maximum	0.8	0.25	0.25	0.59
Endrin	Average ± SD	1.09 ± 0.58	0.62 ± 0.74	0.56 ± 0.33	0.93 ± 0.73
	n.d.; <LOQ; >LOQ	27; 2; 6	35; 3; 1	36; 5; 6	25; 2; 5
	Median	0	0	0	0
	Maximum	1.74	1.73	1.16	2.33
Endosulfan I	Average ± SD	0.38 ± 0.36	0.30 ± 0.25	0.25 ± 0.00	0.38 ± 0.32
	n.d.; <LOQ; >LOQ	4; 26; 5	9; 28; 2	20; 27; 0	6; 21; 5
	Median	0.25	0.25	0.25	0.25
	Maximum	1.96	1.61	0.25	1.71
Endosulfan II	Average ± SD	1.82	n.d.	0.25 ± 0.00	0.25 ± 0.00
	n.d.; <LOQ; >LOQ	34; 0; 1	39; 0; 0	45; 2; 0	28; 4; 0
	Median	0	0	0	0
	Maximum	1.82	0	0.25	0.25
Endosulfan Sulphate	Average ± SD	n.d.	0.25 ± 0.00	0.27 ± 0.08	0.26 ± 0.06
	n.d.; <LOQ; >LOQ	35; 0; 0	37; 2; 0	22; 24; 1	14; 17; 1
	Median	0	0	0.25	0.25
	Maximum	0	0.25	0.63	0.5
Heptachlor	Average ± SD	0.25	0.25 ± 0.00	0.25 ± 0.00	0.25 ± 0.00
	n.d.; <LOQ; >LOQ	34; 1; 0	36; 3; 0	45; 2; 0	29; 3; 0
	Median	0	0	0	0
	Maximum	0.25	0.25	0.25	0.25
Heptachlor Epoxide	Average ± SD	0.25 ± 0.00	0.32 ± 0.20	0.25 ± 0.00	0.25 ± 0.00
	n.d.; <LOQ; >LOQ	26; 9; 0	30; 8; 1	36; 11; 0	22; 10; 0
	Median	0	0	0	0
	Maximum	0.25	0.85	0.25	0.25
pp-DDE	Average ± SD	4.21 ± 4.79	2.23 ± 3.27	1.41 ± 1.68	3.54 ± 3.19
	n.d.; <LOQ; >LOQ	0; 7; 28	0; 13; 26	0; 23; 24	0; 5; 27
	Median	2.4	1.25	0.55	2.92
	Maximum	22.63	19.14	6.84	14.81
op-DDT	Average ± SD	0.25 ± 0.00	0.25 ± 0.00	0.30 ± 0.13	0.46 ± 0.48
	n.d.; <LOQ; >LOQ	20; 15; 0	35; 4; 0	24; 20; 3	6; 19; 7
	Median	0	0	0	0.25
	Maximum	0.25	0.25	0.73	2.36
pp-DDD	Average ± SD	1.34 ± 1.54	0.61 ± 0.63	0.42 ± 0.37	1.06 ± 1.02
	n.d.; <LOQ; >LOQ	7; 12; 16	16; 13; 10	0; 36; 11	0; 10; 22
	Median	0.25	0.25	0.25	0.69
	Maximum	6.2	3.03	1.67	4.69
pp-DDT	Average ± SD	0.66 ± 0.54	0.59 ± 1.11	1.01 ± 1.10	2.06 ± 2.49
	n.d.; <LOQ; >LOQ	12; 12; 11	17; 19; 3	17; 17; 13	5; 7; 20
	Median	0.25	0.25	0.25	0.78
	Maximum	1.79	5.08	3.78	10.87
DDTs Sum	Average ± SD	5.82 ± 6.67	2.95 ± 4.39	2.62 ± 3.12	6.72 ± 6.95
	n.d.; <LOQ; >LOQ	0; 2; 33	0; 6; 33	0; 0; 47	0; 0; 32
	Median	3.36	1.5	3.57	8.73
	Maximum	30.56	24.69	12.57	32.73
α-HCH	Average ± SD	0.25 ± 0.00	0.25 ± 0.00	0.25 ± 0.00	0.25 ± 0.00
	n.d.; <LOQ; >LOQ	29; 6; 0	36; 3; 0	45; 2; 0	29; 3; 0
	Median	0	0	0	0
	Maximum	0.25	0.25	0.25	0.25
β-HCH	Average ± SD	0.25 ± 0.00	0.25 ± 0.00	0.25 ± 0.00	0.25 ± 0.00
	n.d.; <LOQ; >LOQ	30; 5; 0	27; 12; 0	43; 4; 0	24; 8; 0
	Median	0	0	0	0
	Maximum	0.25	0.25	0.25	0.25
γ-HCH (Lindane)	Average ± SD	0.35 ± 0.29	0.32 ± 0.24	0.27 ± 0.07	0.25 ± 0.00
	n.d.; <LOQ; >LOQ	16; 16; 3	19; 18; 2	32; 14; 1	28; 4; 0
	Median	0.25	0.25	0	0
	Maximum	1.41	1.24	0.53	0.25
HCHs Sum	Average ± SD	0.27 ± 0.33	0.26 ± 0.32	0.26 ± 0.06	0.34 ± 0.13
	n.d.; <LOQ; >LOQ	11; 15; 9	14; 16; 9	27; 20; 0	21; 7; 4
	Median	0.25	0.25	0	0
	Maximum	1.66	1.49	0.53	0.5
Chrysene	Average ± SD	n.d.	0.50 ± 0.00	0.50 ± 0.00	0.50 ± 0.00
	n.d.; <LOQ; >LOQ	35; 0; 0	37; 2; 0	19; 28; 0	15; 17; 0
	Median	0	0	0.5	0.5
	Maximum	0	0.5	0.5	0.5
Benz(a)anthracene	Average ± SD	0.78 ± 0.39	0.50 ± 0.00	0.50 ± 0.00	0.50 ± 0.00
	n.d.; <LOQ; >LOQ	30; 3; 2	25; 14; 0	7; 40; 0	10; 22; 0
	Median	0	0	0.5	0.5
	Maximum	1.31	0.5	0.5	0.5
Benzo(b)fluoranthene	Average ± SD	0.76 ± 0.37	0.80 ± 0.39	0.69 ± 0.12	0.77 ± 0.07
	n.d.; <LOQ; >LOQ	33; 1; 0	34; 2; 3	42; 1; 4	27; 0; 5
	Median	0	0	0	0
	Maximum	1.03	1.44	0.83	0.84
Benzo(a)pyrene	Average ± SD	0.5	0.5	0.5	0.5
	n.d.; <LOQ; >LOQ	34; 1; 0	38; 1; 0	39; 8; 0	31; 1; 0
	Median	0	0	0	0
	Maximum	0.5	0.5	0.5	0.5
PAHs Sum	Average ± SD	0.74 ± 0.34	0.70 ± 0.37	0.94 ± 0.36	0.88 ± 0.33
	n.d.; <LOQ; >LOQ	27; 5; 3	21; 12; 6	3; 13; 21	5; 9; 18
	Median	0	0	1	1
	Maximum	1.31	1.94	1.76	1.84

n.d. = not detected.

## Data Availability

The data presented in this study are available on request from the corresponding author.
